# The reasons why fractional flow reserve and instantaneous wave-free ratio are similar using wave separation analysis

**DOI:** 10.1186/s12872-021-01855-4

**Published:** 2021-01-25

**Authors:** Soohong Min, Gwansuk Kang, Dong-Guk Paeng, Joon Hyouk Choi

**Affiliations:** 1 Department of Ocean System Engineering, Jeju National University, Jeju, Korea; 2 Division of Gastroenterology and Hepatology, Stanford University School of Medicine, Stanford, CA USA; 3 Department of Radiology and Medical Imaging, School of Medicine, University of Virginia, Charlottesville, VA USA; 4 Department of Cardiology, Jeju National University School of Medicine, Jeju National University Hospital, Jeju, Korea

**Keywords:** Wave intensity analysis (WIA), Fractional flow reserve (FFR), Instantaneous wave-free ratio (IFR), Coronary artery, Wave separation analysis (WSA)

## Abstract

**Background and objectives:**

Fractional flow reserve (FFR) and instantaneous wave-free ratio (iFR) are the two most commonly used coronary indices of physiological stenosis severity based on pressure. To minimize the effect of wedge pressure (P_*wedge*_), FFR is measured during hyperemia conditions, and iFR is calculated as the ratio of distal and aortic pressures (P_*d*_/P_*a*_) in the wave-free period. The goal of this study was to predict P_*wedge*_ using the backward wave (P_*back*_) through wave separation analysis (WSA) and to reflect the effect of P_*wedge*_ on FFR and iFR to identify the relationship between the two indices.

**Methods:**

An in vitro circulation system was constructed to calculate P_*wedge*_. The measurements were performed in cases with stenosis percentages of 48, 71, and 88% and with hydrostatic pressures of 10 and 30 mmHg. Then, the correlation between P_*back*_ by WSA and P_*wedge*_ was calculated. In vivo coronary flow and pressure were simultaneously measured for 11 vessels in all patients. The FFR and iFR values were reconstructed as the ratios of forward wave at distal and proximal sites during hyperemia and at rest, respectively.

**Results:**

Based on the in vitro* results*, the correlation between P_*back*_ and P_*wedge*_ was high (r = 0.990, *p* < 0.0001). In vivo results showed high correlations between FFR and reconstructed FFR (r = 0.992, *p* < 0.001) and between iFR and reconstructed iFR (r = 0.930, *p* < 0.001).

**Conclusions:**

Reconstructed FFR and iFR were in good agreement with conventional FFR and iFR. FFR and iFR can be expressed as the variation of trans-stenotic forward pressure, indicating that the two values are inferred from the same formula under different conditions.

## Background

Fractional flow reserve (FFR) is considered the “gold standard” among invasive physiological diagnostic methods for determining the percutaneous coronary intervention of intermediate lesions in patients with stable angina [1]. Therefore, FFR was used as a comparative group for instantaneous wave-free ratio (iFR) in some studies. In these studies, iFR has been reported to be faster, less uncomfortable, and not inferior compared to FFR [2, 3].

To explain FFR theoretically, coronary wedge pressure (P_*wedge*_) is a very important factor. FFR is measured when P_*wedge*_ is minimized by pharmacological hyperemia [4]. The P_*wedge*_ wave is characterized by rapid decline in and formation of baseline in pre-systole [5]. This event is explained by backward-propagating suction-waves in wave intensity analysis (WIA) and loss of the Windkessel effect due to occlusion [6, 7]. The Windkessel effect is defined as the condition where the pressure does not fall to zero due to capacitive elements and resistance [8]. If iFR can also be explained by P_*wedge*_ or backward wave through wave separation analysis (WSA), we can explain how the two indices are similar or different. Thus, iFR could be measured when P_*wedge*_ is minimized during the wave-free period of diastole without hyperemia.

This study was based on the assumption that coronary pressure waves can be separated into constituent forward (P_*for*_) and backward (P_*back*_) waves using WSA. We attempted to prove this assumption as follows: (1) P_*back*_ can replace P_*wedge*_ from an in vitro experimental study; and (2) FFR and iFR can be reconstructed using P_*back*_ obtained from WSA and compared with conventional FFR and iFR from in vivo measurement results. This study may be the first to identify similarities and differences between FFR and iFR using WSA.

## Experiment and method

### Theoretical background

The iFR is defined as the ratio of distal pressure (P_*d*_) to aortic pressure (P_*a*_) at rest during a wave-free period, as shown in Eq. 1:

1 $${\text{iFR}} = \frac{{{\text{P}}_{d} }}{{{\text{P}}_{a} }}\,at\,rest\,during\,wave{-}free\,period$$

**Fig. 1:**
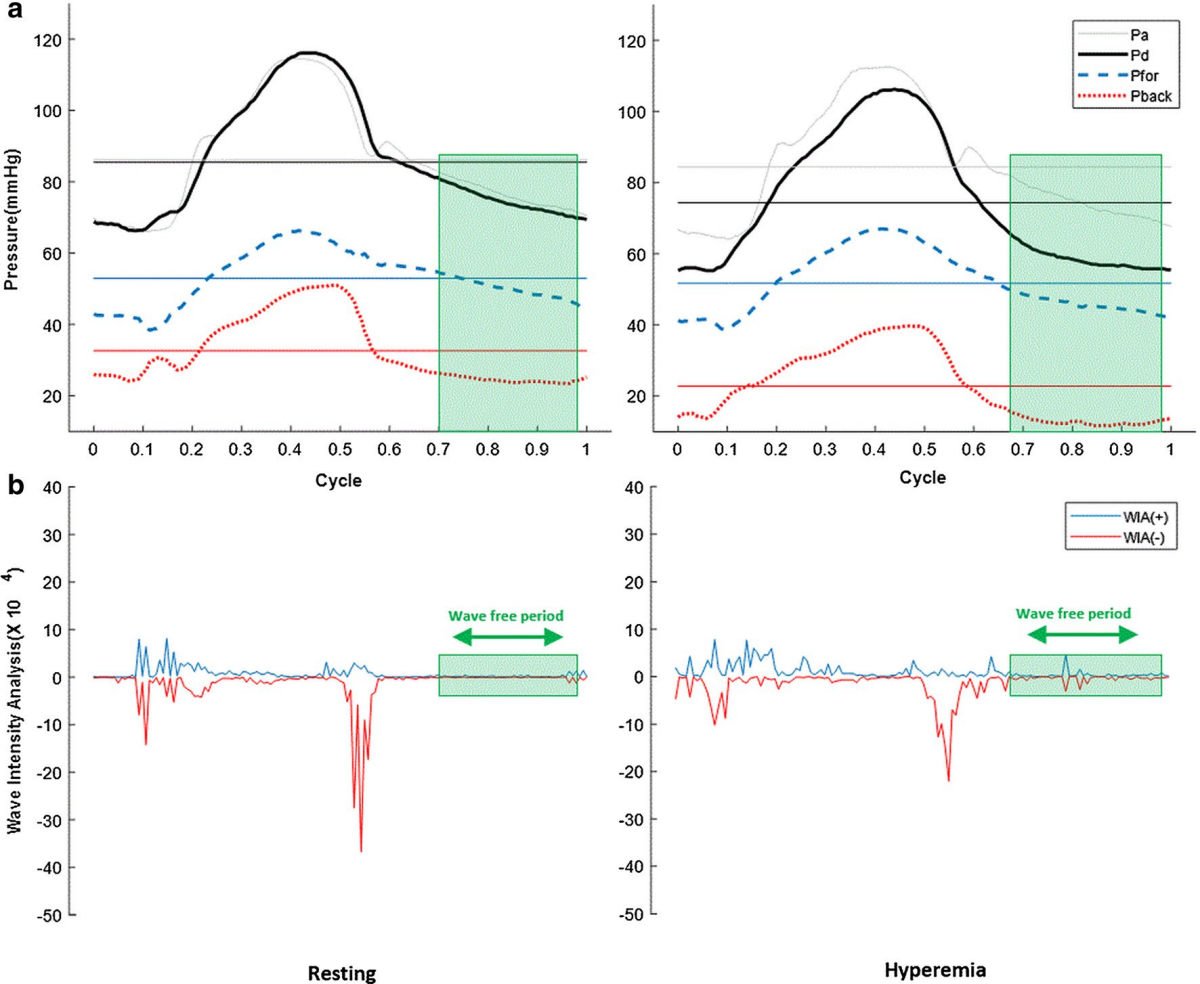
Changes in pressures before and after Hyperemia and wave free period extraction through WIA analysis. **a** Aortic and distal pressures (P_*a*_, P_*d*_), forward and backward waves (P_*for*_, P_*back*_) in a cycle at pre-hyperemia and hyperemia, and horizontal lines are their average values over a cycle. P_*a*_ decreased little but P_*d*_ decreased more at hyperemia. Although overall P_*back*_ decreases a lot at hyperemia, there is little change in P_*for*_ between pre-hyperemia and hyperemia. **b** Wave free period was extracted through wave intensity analysis (WIA) as shown in green boxes

Assuming that P_*wedge*_ or ${\text{P}}_{back} + {\text{P}}_{static}$ is baseline in the wave-free period, the slopes of P_*d*_ and P_*a*_, and ${\text{P}}_{for} \left( {prox} \right)$ and ${\text{P}}_{for} \left( {dist} \right)$ in pre-systolic phase could be the same, as shown in Fig. 1. Thus, reconstructed iFR is redefined as follows in Eq. 2:

2 $${\text{reconstructed iFR}} = \frac{{{\text{P}}_{d} - \left( {{\text{P}}_{back} + {\text{P}}_{static} } \right)}}{{{\text{P}}_{a} - \left( {{\text{P}}_{back} + {\text{P}}_{static} } \right)}} \approx \frac{{{\text{P}}_{for} \left( {dist} \right)}}{{{\text{P}}_{for} \left( {prox} \right)}} \left( {at\,resting} \right)$$

Reconstructed iFR is calculated as the average pressure of an entire cycle at rest and is not the same as conventional iFR, which is determined only in the wave-free period. Reconstructed iFR, which is the ratio of P_*for*_ at distal and proximal locations averaged over a whole cycle, is assumed to be similar to conventional iFR. This assumption will be shown to be appropriate by in vitro and in vivo experimental results in this study.

The corresponding FFR of the coronary artery ($FFR_{cor}$) is overestimated based on central vein pressure (P_*v*_) instead of P_*wedge*_ considering collateral flow [4]. $FFR_{cor}$ is defined as the ratio of distal pressure (P_*d*_) and aortic pressure (P_*a*_) when the effect of P_*wedge*_ is subtracted at hyperemia as shown in Eq. 3:

3 $$FFR_{cor} = \frac{{{\text{P}}_{d} - {\text{P}}_{wedge} }}{{{\text{P}}_{a} - {\text{P}}_{wedge} }} \left( {at\,hyperemia} \right)$$

Reconstructed FFR is the ratio of P_*for*_ at the distal and proximal locations when P_*wedge*_ is assumed to be ${\text{P}}_{back} + {\text{P}}_{static}$ as shown in Eq. 4:

4 $${\text{reconstructed FFR}} = \frac{{{\text{P}}_{d} - \left( {{\text{P}}_{back} + {\text{P}}_{static} } \right)}}{{{\text{P}}_{a} - \left( {{\text{P}}_{back} + {\text{P}}_{static} } \right)}} = \frac{{{\text{P}}_{for} \left( {dist} \right)}}{{{\text{P}}_{for} \left( {prox} \right)}} \left( {at\,hyperemia} \right)$$

WIA was performed to obtain wave-free periods using representative flow speed (U) and pressure (P) as in Eqs. 5 and 6:

5 $${\text{WI}}(+) = \frac{1}{4\rho c}\left( {\frac{dP}{{dt}} + \rho c\frac{dU}{{dt}}} \right)^{2}$$

6 $${\text{WI}}(-) = \frac{1}{4\rho c}\left( {\frac{dP}{{dt}} - \rho c\frac{dU}{{dt}}} \right)^{2}$$

where ρ is the density of blood [1050 kg/m^−3^], and c is wave speed [m/s] calculated using the single-point equation. The wave-free period is defined as the time from WI (-) = 0 to the end of diastole for 5 ms [7].

**Fig. 2:**
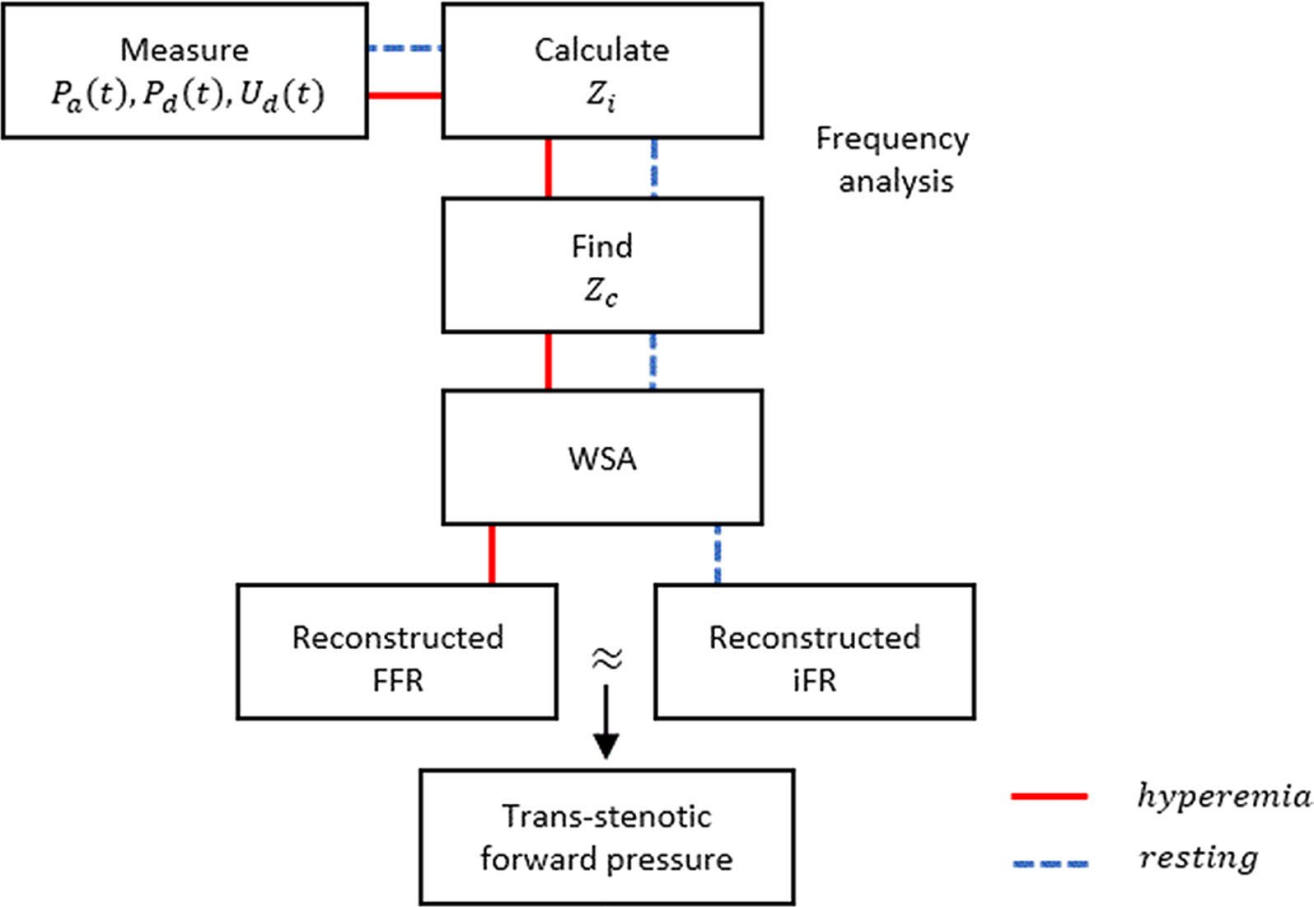
Schematic diagram of the process of calculating reconstructed FFR and iFR. Measure P_*a*_, P_*d*_, U_*d*_, divide P_*d*_ by U_*d*_, calculate Z_*i*_, and find Z_*c*_ through frequency analysis of Z_*i*_. P_*back*_ is calculated through WSA. Reconstructed FFR and iFR are calculated by the same formula using trans-stenotic forward pressure at hyperemia and rest conditions, respectively

WSA was performed to obtain P_*for*_ and P_*back*_ using representative flow (F(t)) and pressure (P(t)) obtained from Eqs. 7 and 8:

7 $${\text{P}}_{for} \left( t \right) = \frac{{\left[ {P\left( t \right) + Z_{c} \times F\left( t \right)} \right]}}{2}$$

8 $${\text{P}}_{back} \left( t \right) = \frac{{\left[ {P\left( t \right) - Z_{c} \times F\left( t \right)} \right]}}{2}$$

where Z_*c*_ is characteristic impedance and is defined as an input impedance (Z_*i*_) in the absence of wave reflection. Z_*i*_ is defined as resistance or impedance obtained by frequency analysis of representative pressure and blood flow using Fourier analysis [9]. At the same time, the modulus (division) and phase (subtraction) of impedance were automatically calculated. Therefore, the impedance modulus at zero frequency (0-impedance) is mean pressure/mean flow. There are many methods of obtaining Z_*c*_. In general, Z_*c*_ is defined as the modulus at the zero crossing point or a point close to zero in phase. The reason for this distinction is that the negative phase is the imaginary component of Fourier analysis. Previous studies have addressed Z_*c*_ with a fixed frequency [9–15]. However, Z_*c*_ can be changed depending on the situation [9]. In this study, we used flexible Z_*c*_, which is defined as the average modulus of four harmonics of the fundamental frequency after zero crossing or close to zero in phase less than 10 Hz. The process of calculating the reconstructed FFR and iFR is briefly shown in Fig. 2.

### In vitro coronary artery circulation system

**Fig. 3:**
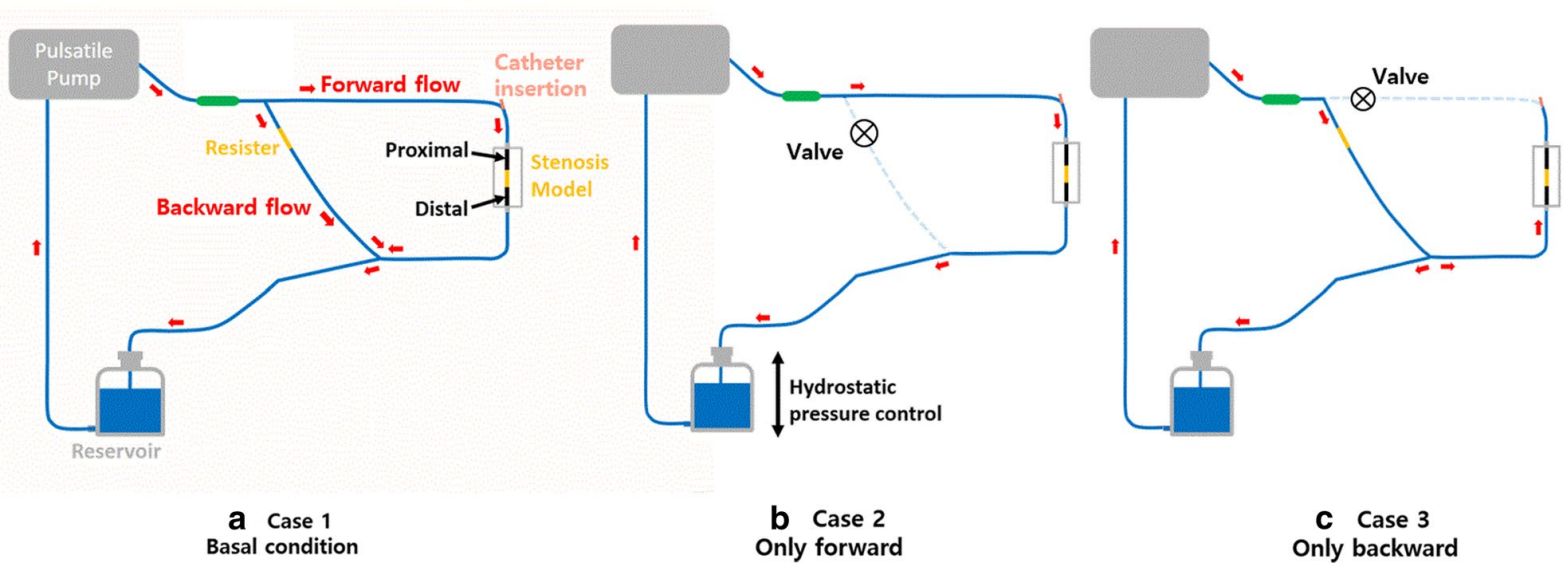
An in vitro coronary circulation system. Three conditions were created as (a–c). **a** Basal condition, in which both forward and backward flows existed. Resister was used to control the ratio of forward and backward flow, and the pressure and blood flow were stabilized using the Windkessel model. **b** Only forward condition, in which backward flow was blocked. By adjusting the height of the reservoir, the P_*static*_ was controlled. High P_*static*_ is assumed to be pre-hyperemia and low P_*static*_ is assumed to be hyperemia. **c** Only backward flow condition, in which forward flow was blocked for measuring

In this study, we designed an in vitro coronary blood circulation system. As shown in Fig. 3, a catheter (Combo Wire XT ®, Volcano Corporation, San Diego, CA, USA) was inserted into a tube to simultaneously measure pressure and flow speed at the proximal and distal sites of stenosis. A pulsatile pump (Model 55–3305, Harvard Apparatus Corp., USA) was used to mimic heart motion. The pump rate was fixed to 60 rotations/min, and the operative phase ratio (OPR; systolic time over a cyclic time) was set to 60%. The tube was filled with 1.5 L of Doppler fluid (Model 707, ATS Laboratories, USA). The viscosity of this fluid (5 cP) is similar to that of human blood. The tube was an IXAK® silicon tube (SL-0710, TOMMYHECO, KOREA) with an internal diameter of 5 mm. To reflect stenotic coronary arteries in the system, stenotic vessels of 48, 71, and 88% (minimum vessel area/maximum vessel area) were created using a three-dimensional printer. The minimal luminal dimensions of each model were 28, 46, and 64%. A Windkessel model was constructed using an air tank to control blood flow, pressure waveforms, and phase differences, which were similar to those observed in the human coronary artery. This approach can eliminate negative pressure and exerts zero flow on the system. Measurements were performed at 20 mm proximal to the stenosis site, and a catheter was inserted 200 mm proximal to the site of stenosis.

We created three conditions. Figure 3a is the basal condition. There is a combination of forward and backward pressures in the coronary artery. Resistance with stenosis can be used to control the ratio of forward and backward flow. By adjusting the inner diameter of the resistor, the amount of fluid directed to the stenotic phantom can be adjusted. Therefore, the ratio of forward and backward flow can be controlled, which makes it possible to reproduce a human-like automatic control ability of the blood flow. If the inner diameter of the resistor is larger than that of each site of stenosis, a phenomenon occurs where the backward flow is larger than the forward flow. Forward and backward pressures were separated using WSA in this condition. The reservoir was used to control P_*static*_ in the blood flow system, which was adjusted by varying the height.

Figure 3b is a condition of forward flow only without backward flow. High P_*static*_ and low P_*static*_ values are assumed to represent pre-hyperemia and hyperemia conditions, respectively. P_*d*_/P_*a*_ values measured in pre-hyperemia and hyperemia conditions were assumed to indicate reconstructed iFR and reconstructed FFR, respectively.

To confirm that this in vitro circulatory system mimics the blood flow of the coronary system, the flow speed of Case 2 (only forward condition) was divided by that of Case 1 (basal condition), and the ratio was compared to the coronary flow reserve.

Figure 3c depicts the P_*wedge*_. In clinical practice, P_*wedge*_ is measured at a distal site when the artery is blocked with a balloon. To reflect this in the in vitro system, we blocked the branch point toward the coronary phantom through the valve. The measured pressure in this case was compared with the backward pressure that was calculated using WSA.

### In vivo experiment

The study protocol was approved by the institutional review board of Jeju National University Hospital (2016-07-011).

Coronary angiography and pressure-flow measurements were obtained using standard techniques [16]. Angiographic views were obtained following administration of intracoronary nitrate in all cases (200 or 300 µg). We used 0.014-inch pressure and Doppler sensor-tipped wires (ComboWire XT, Volcano Corporation, San Diego, CA, USA). The distal pressure was set to zero and equalized to the aortic pressure at the coronary ostium before being positioned at least three vessel diameters distal to the site of stenosis. Adenosine was administered for hyperemia by intravenous infusion based on 11 measurements (140 µg/kg/min). When a ComboWire was used, the electrocardiogram, pressures, and flow velocity signals were directly extracted from the digital archive of the device console (ComboMap, Volcano Corporation). Data were analyzed off-line, using a custom software package designed by Labview (National Instruments, Austin, TX, USA). Stenosed vessels were defined as vessels that had an angiographically visible stenosis from 40 to 70% severity, as determined visually by an operating physician at the time of coronary angiography.

Resting indices were calculated at a time of stability, allowing for a return to stable baseline conditions after any preceding injection of contrast or saline. Hyperemic indices were determined during stable hyperemia, excluding cases with ectopy or conduction delay. Representative flow and pressure waves were obtained by an average method using recordings of 5–15 consecutive cycles both at rest and during hyperemia. These procedures were necessary to achieve linearity and time invariance. FFR and iFR were calculated as the ratio of mean P_*d*_ to P_*a*_ at hyperemia during a whole cycle and at rest during a wave-free period, respectively.

The reconstructed FFR and reconstructed iFR were calculated by the following equation of $\left( {{\text{P}}_{d} - {\text{P}}_{back} } \right)/\left( {{\text{P}}_{a} - {\text{P}}_{back} } \right)$ at hyperemia and at rest conditions, respectively.

### Statistical analysis

All statistical analyses were performed using the Statistical Package for the Social Sciences (SPSS), version 23.0 software (SPSS Statistics for Macintosh, IBM Corp. Armonk, NY, USA). The values of continuous variables are mean and standard deviation (SD), and categorical variables are expressed as frequency and percentage. The comparison of continuous variables between groups was performed using the independent sample t-test, and the categorical variables were assessed with a chi-square test. The correlation analysis between groups was performed by simple correlation analysis. For each statistic, the significance level was less than 0.05.

## Result

Parts of this study were presented to the ACC.19: The American College of Cardiology 68th Annual Scientific Sessions, New Orleans, USA, March 16–18, 2019 [17, 18].

### In vitro experiment

A total of 18 cases were analyzed according to stenoses (48, 71, and 88%), and P_*static*_ (10 and 30 mmHg) values obtained from mock circulatory experiments. The measured and calculated data are summarized in Table 1.

**Table 1:** Measured pressure and distal flow speed in the 3 models of stenosis

Stenosis (%)	P_*static*_ (mmHg)	Case 1					Case 2			Case3
		Basal condition					Forward flow condition			Wedge condition
		Observed indices			Calculated indices		Observed indices			
		P_*a*_ (mmHg)	P_*d*_ (mmHg)	Distal flow (cm/s)	P_*for*_ (mmHg)	P_*back*_ (mmHg)	P_*a*_ (mmHg)	P_*d*_ (mmHg)	Distal flow (cm/s)	P_*wedge*_ (mmHg)
48	10	20.2	18.2	13.98	10.9	7.3	23.27	18.83	29.9	19.7
48	30	40.6	40.4	16.40	27.8	12.6	44.50	40.29	31.9	40.1
71	10	21.7	19.3	23.53	11.3	7.0	25.30	16.14	37.7	15.0
71	30	43.1	41.2	24.76	29.3	12.0	46.64	41.26	37.7	40.1
88	10	36.6	18.5	29.46	11.8	6.7	44.62	20.51	35.4	19.3
88	30	57.0	38.4	30.92	29.0	9.5	64.05	38.45	37.4	38.4

P_*a*_, coronary aortic pressure; P_*d*_, coronary distal pressure; P_*back*_, backward pressure; P_*for*_, forward pressure; P_*static*_, hydro static pressure; P_*wedge*_, coronary wedge pressure

When the static pressure was 10 mmHg, the distal flow ratio (only forward flow/basal flow) according to stenosis increased to 2.2, 1.5, and 1.2 as the stenosis increased to 49, 71, and 88%, respectively. When the static pressure was 30 mmHg, the distal flow ratios ​were 2.5, 1.6, and 1.2 at stenosis rates of 49, 71, and 88%, respectively.

The $P_{d} /P_{a}$ ratio for Case 1 decreased in the order of stenosis (48, 71, and 88%) at each P_*static*_ (10 mmHg, 0.81, 0.64, 0.46; 30 mmHg, 0.91, 0.88, 0.60, respectively). The distal flow ratio in high P_*static*_ was higher than that in low P_*static*_.

**Fig. 4:**
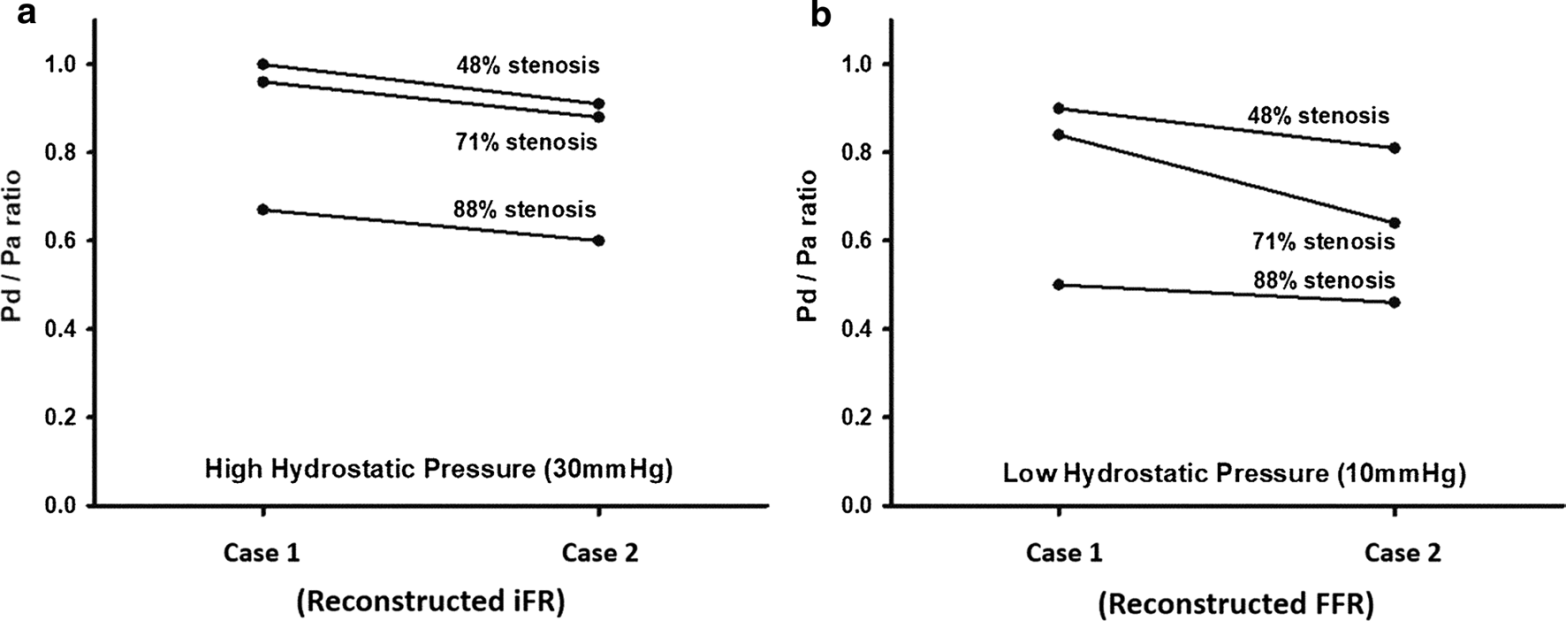
P_*d*_*/*P_*a*_ in Case1 and 2 at 3 stenosis when P_*static*_ was 30 and 10 mmHg. When the static pressure was 30 mmHg(High hydrostatic pressure), P_*d*_*/*P_*a*_ in case 2 was set as reconstructed iFR, and when the static pressure was 10 mmHg(Low hydrostatic pressure), P_*d*_*/*P_*a*_ in case 2 was set as reconstructed FFR

The $P_{d} /P_{a}$ ratio for Case 2 decreased in the order of stenosis (48, 71, and 88%) at each P_*static*_ (10 mm Hg, 0.90, 0.84, and 0.5; 30 mmHg, 1.00, 0.96, and 0.67, respectively). The change in $P_{d} /P_{a}$ between Case 1 and Case 2 was larger for low P_*static*_ than for high P_*static*_, as shown in Fig. 4.

**Fig. 5:**
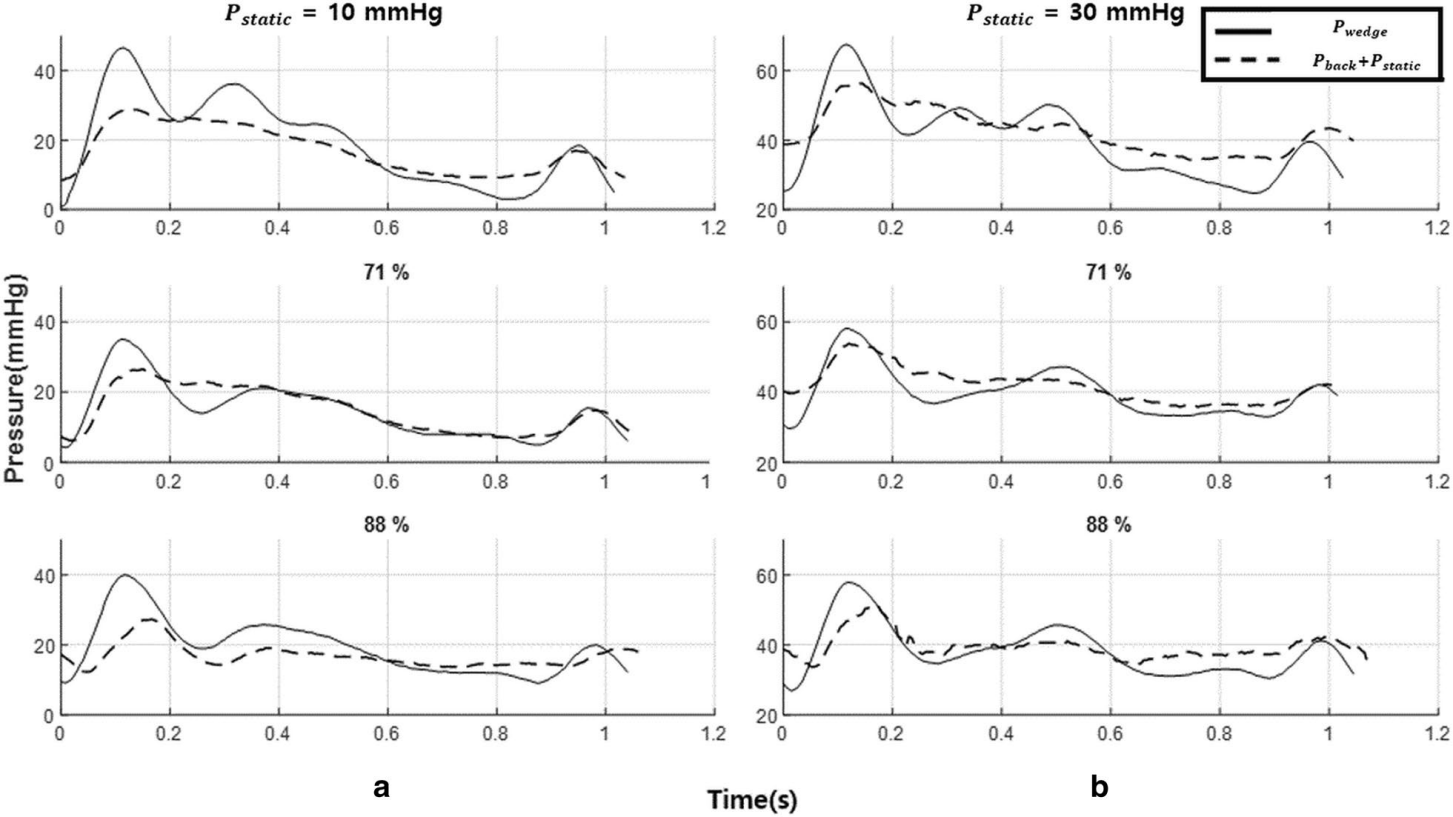
The waveform of P_*wedge*_ and P_*back*_. The static pressure was **a** 10 and **b** 30 mmHg at stenosis 48%. Each static pressure was added to P_*back*_

**Fig. 6:**
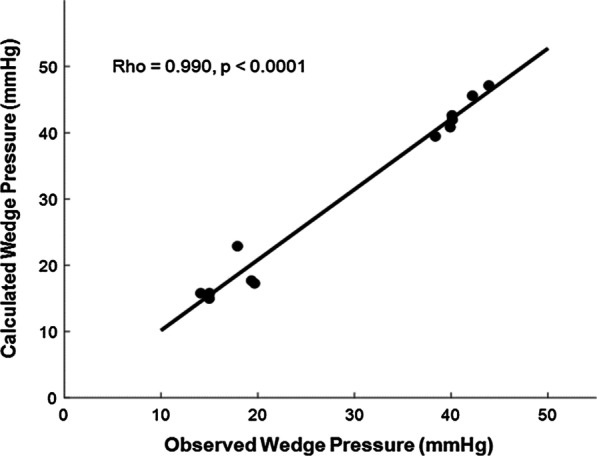
The correlation between observed wedge pressure (P_*wedge*_) and calculated wedge pressure P_*back*_ + P_*static*_. The correlation was high (r = 0.990, *p* < 0.0001) and slope was 1.0612

The waveforms and magnitude of the observed P_*wedge*_ and ${\text{P}}_{back} + {\text{P}}_{static}$ were very similar (Fig. 5). This trend was also observed in other cases. P_*wedge*_ always contains static pressure. Correlation with the ${\text{P}}_{back} + {\text{P}}_{static}$ and P_*wedge*_ was high (r = 0.990, *p* < 0.0001, Fig. 6), and the slope was 1.0612.

### In vivo experiment

Nine patients in whom we were able to simultaneously measure blood flow and blood pressure in the proximal and distal regions were compared with pre- and post-hyperemia values of distal forward pressure P_*for*_ and distal backward pressure P_*back*_ using Z_*c*_ in 11 coronary blood vessels. The results are summarized in Table 2.

**Table 2:** Pressures (mmHg) at proximal and distal site at rest and hyperemia, and indices obtained from eleven coronary vessels in this study

Patients	Vessels	FFR	iFR	P_*a*_	P_*d*_	P_*a*_	P_*d*_	P_*for*_ (distal)	P_*back*_ (distal)	P_*for*_ (distal)	P_*back*_ (distal)	Reconstructed FFR	Reconstructed iFR
				Resting		Hyperemia		Resting		Hyperemia			
1	LAD	0.75	0.92	74.9	69.1	63.4	49.8	48.1	21	40.2	9.6	0.75	0.89
2	LAD	0.8	0.94	88.1	83.1	77.7	63.8	57.8	25.3	57.1	6.7	0.80	0.92
3	LAD	0.85	0.89	120.4	113.8	103.6	93.3	80.8	33	78.2	15.1	0.88	0.93
4	LAD	0.87	0.97	126.6	119.3	106.9	96.1	73.5	45.8	68.5	27.6	0.86	0.91
4	LCx	0.96	1.04	119.8	119.8	109.5	106.8	84.9	37.9	90	16.9	0.97	1.00
4	RCA	0.96	1.07	123.4	128.3	94.7	107.1	88.6	39.7	79.3	27.8	0.97	1.06
5	Ramus	0.94	0.99	102.5	101.3	109.5	90.8	60.2	40.1	70.3	20.5	0.95	0.98
6	LAD	0.73	0.82	109.7	96.0	100.8	80.2	70.3	25.7	68.6	11.6	0.70	0.84
7	LAD	0.8	0.98	108.8	104.5	80.9	87.2	62.4	42.1	59.2	28	0.81	0.94
8	LAD	0.66	0.74	88.1	67.6	80.9	57.4	39.6	28	45.2	12.2	0.66	0.66
9	LAD	0.89	1.07	105.7	108.3	98.8	90.3	63.7	44.6	75.1	15.2	0.90	1.04

FFR, fractional flow reserve; iFR, instantaneous wave-free ratio; P_*a*_, coronary aortic pressure; P_*d*_, coronary distal pressure; P_*back*_, backward pressure; P_*for*_, forward pressure

**Fig. 7:**
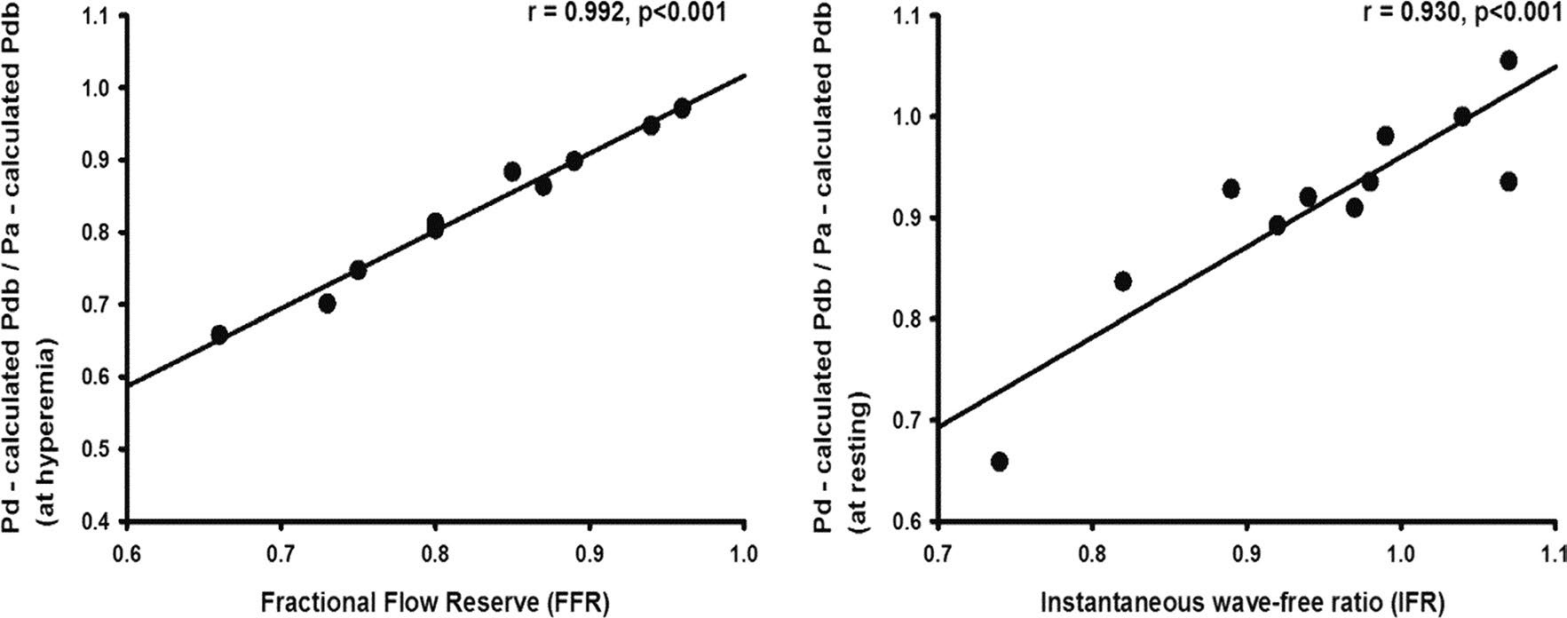
Correlation between **a** FFR and reconstructed FFR, **b** iFR and reconstructed iFR. Both graphs show a high correlation

The correlations between conventional FFR and reconstructed FFR and between conventional iFR and reconstructed iFR were positive (r = 0.992, *p* < 0.001 and 0.930, *p* < 0.001, respectively; Fig. 7).

## Discussion

In this study, coronary pressure waves could be separated into constituent forward (P_*for*_) and backward (P_*back*_) waves through WSA using frequency analysis. It could be said that P_*back*_ reflected P_*wedge*_ without P_*static*_ experimentally. It was shown that $FFR_{cor}$ and iFR could be expressed in trans-stenotic ΔP_*for*_ either with or without hyperemia, which indicated that the two indices were inferred by removing P_*wedge*_ or P_*back*_. In vivo*,* $FFR_{cor}$ and iFR were reconstructed assuming that the P_*back*_ and P_*wedge*_were similar. The reconstructed indices were highly correlated with the conventional ones. Therefore, to our knowledge, this study is the first to identify similarities and differences between $FFR_{cor}$ and iFR using WSA.

### Theoretical background of FFR and iFR through WSA

In this study, P_*back*_ was characterized as undergoing rapid decline and forming baseline observed during pre-systole either with or without hyperemia. This finding is similar to the characteristics of P_*wedge*_ [5]. After forming the baseline of P_*back*_, the slope of P_*for*_ was similar to the slope of coronary pressure. The period of forming the baseline of P_*back*_ was similar to the wave-free period. Eventually, the amplitude of P_*for*_ was smaller than the amplitude of coronary pressure (Fig. 1). During the wave-free period, P_*a*_, P_*d*_, and P_*for*_ could have the same slope because P_*back*_ for ms the baseline. The ratio between the lines with the same slope may be different, but the value in that interval is constant. iFR is defined as P_*d*_/P_*a*_ in the wave-free interval. Therefore, iFR may be related to P_*for*_(distal)/P_*for*_(proximal) during the wave-free period. Furthermore, as the amplitude of P_*for*_ without P_*back*_ is low, the mean P_*for*_ of the whole cycle and the mean P_*for*_ of the wave-free period may be similar as a factor of ratio. As a result, in this study, reconstructed iFR was defined as P_*for*_(distal)/P_*for*_(proximal) in Eq. 1. The reconstructed and conventional iFRs showed a good correlation based on in vivo results.

During hyperemia, the theoretical FFR of the coronary artery $(FFR_{cor} )$ is $(P_{d} - P_{w} )/(P_{a} - P_{w} )$, while the FFR of the myocardium $(FFR_{myo} )$ is $(P_{d} - P_{v} )/(P_{a} - P_{v} )$, where $P_{v}$ represents the mean central venous pressure [4]. The FFR is the ratio between mean values. A mean value is decreased when both the peak and the baseline are lowered. In this study, hyperemia mainly reduced the baseline of pressure (Fig. 1). Moreover, P_*back*_ was not zero but still decreased during hyperemia, and P_*for*_ was constant under the Windkessel effect.

The difference between $FFR_{cor}$ and $FFR_{myo}$ is described by collateral flow [4]. P_*wedge*_ is closely related to the collateral flow [19]. In addition, hyperemia theoretically reflects the offset of P_*wedge*_ and $P_{v}$ in the conventional FFRs [4]. However, the values of the P_*wedge*_ or $P_{v}$ would not be practically removed in hyperemia.

The FFR is based on the assumption that resistances both with and without stenosis are the same. Without collateral flow, this assumption implies that FFRmyo progressively overestimates the FFR using flow with increasing stenosis severity [4]. Thus, an attempt has been made to overcome this mismatch in reconstructing the FFR using zero flow pressure ($P_{zf}$). The formula is as follows: ${\text{FFR}} = (P_{d} - P_{zf} )/(P_{a} - P_{zf} )$. FFR using $P_{zf}$ was in good agreement with the FFR using flow [20]. Because of the diastolic characteristics of the coronary arteries, $P_{zf}$ is independent of contraction and auto-regulation, showing conductance of the vessels and pure resistance [21–23]. However, P_*wedge*_ is generally smaller than $P_{zf}$ due to the non-linearity of the pressure-flow relationship and existence of cardiac contraction either with or without collateral flow [23–25]. Conceptually, P_*back*_ by WSA was similar to $P_{zf}$ in this study. This means that both FFR and iFR could be trans-stenotic ΔP_*for*_, which can be expressed using the same formula, although their methods are different (Eqs. 1 and 2).

### Difference between FFR and iFR

In order to replace the FFR using flow with FFR using pressure, hyperemia is required to offset P_*wedge*_ and P_*v*_ [4]. As mentioned above, the reconstructed iFR was calculated by subtracting P_*back*_ at rest, which is assumed to be P_*wedge*_. Theoretically, P_*for*_ can be determined by the stroke volume, which is related with inflow, resistance, compliance, and volume capacity, because the Windkessel effect is observed and systolic resistance by subtracting P_*for*_ is absent [8]. It is similar to systemic circulation. When administered for hyperemia, adenosine is reported to have little effect on the stroke volume or ejection fraction [26]. There is no significant change in blood volume without bleeding. Therefore, the difference in P_*for*_ with or without hyperemia is mainly dependent on resistance. The change of resistance according to the situation from rest to hyperemia could be the change of P_*static*_ or P_*v*_. Thus, the difference between iFR and FFR is likely to be the difference of P_*for*_ in relation to P_*static*_ or P_*v*_ rather than P_*wedge*_ or P_*back*_.

As myocardium oxygen consumption (MVO_2_) increases due to enlargement of micro-vessels, resistance is reduced, and flow is increased. This trend is mainly regulated by the adenosine and nitric oxide (NO) metabolites in the myocardium. In the presence of significant stenosis, the role of adenosine may be activated in micro-vessels, so the reactivity of hyperemia by adenosine may be lowered. In other words, resistance due to pharmacological hyperemia may be smaller in significant stenosis than in nonsignificant stenosis [27, 28].

### Clinical implications and future studies

This is the first paper to prove that iFR and FFR are theoretically related using WSA up to our knowledge. The incidence of clinically appropriate hyperemia is not well known. In fact, it is difficult to verify hyperemia even with constant drug increases or drug changes. Thus, nonsignificant changes of P_*for*_ during hyperemia may be explained by the limitations of the assumption of constant resistance either with or without stenosis in FFR, and pharmacological hyperemia with inappropriate offsets of P_*wedge*_ and P_*v*_. Nevertheless, this study assumes that P_*for*_ is the primary factor for determining iFR and FFR using pressure. This assumption was confirmed by in vivo* and *in vitro results. Theoretically, the wave free period for iFR was made by WIA. The slope of P_*for*_ by WSA in the wave free period was similar to that of P_*a*_ and P_*d*_ in this period. Therefore, it will be possible to create a new algorithm of the wave free period for iFR.

### Limitation

In this paper, we tried to reflect the characteristics of various coronary arteries such as blood flow and pressure waveforms, in the human body. There are many differences in blood flow and pressure waveforms in human coronary arteries. However, this variation did not pose a problem because we used the average values for pressure and blood flow.

It cannot be said that P_*back*_ reflects P_*wedge*_ experimentally. The constituent waves from WSA are the estimated values [10]. Moreover, the purpose of this study was to prove that iFR and FFR share the same formula. Therefore, the most important factors are morphological pattern and phase; acquiring accurate values was not the main goal. Accordingly, several trials of WSA were performed considering different Z_*c*_ values. The results from various trials of WSA showed a similar pattern.

Z_*c*_ increased during hyperemia [14]. However, Z_*c*_ decreased in this study. Although this result cannot be explained, it is inferred that there are differences in the species or drugs used for hyperemia. To verify this hypothesis, additional experiments for Z_*c*_ will be needed.

The combo wire we used could measure pressure and flow at the catheter tip where Pd was measured. However, when measuring Pa, the flow rate was not measured. Measurements in the proximal region were also not performed. So we could not calculate the proximal forward pressure using WSA. In a future study, the pressure and flow of the proximal site will be measured to check the separated pressure of the proximal and distal sites.

When measuring pressure and flow, we extracted one averaged cycle from more than 5 cycles based on the ECG signal. Groups with atrial fibrillation and other arrhythmia were excluded from the analysis. The process of finding Z_*c*_ for WSA may vary from person to person. Therefore, we made a program through Labview software to minimize the error of observers. Three investigators calculated the WSA using the software, and there was little error although the accuracy was not quantified.

## Conclusions

In this study, we calculated P_*back*_ in the coronary artery using WSA and confirmed that the correlation between P_*back*_ and P_*wedge*_ was high. The FFR and iFR were reconstructed by reflecting P_*wedge*_ calculated through P_*back*_. It could be proved deductively that FFR and iFR can be expressed in the trans-stenotic ΔP_*for*_. Therefore, the two indices are inferred from the same formula under different conditions. Similarities and differences between iFR and FFR were thus confirmed.

## Data Availability

The datasets used and/or analyzed during the current study are available from the corresponding author on reasonable request.

## Abbreviations

FFR: Fractional flow reserve
iFR: Instantaneous wave-free ratio
P_*wedge*_: Wedge pressure
WSA: Wave separation analysis
WIA: Wave intensity analysis
P_*for*_: Forward pressure
P_*back*_: Backward pressure
P_*d*_: Distal pressure
P_*for*_: Aortic pressure
P_*static*_: Static pressure
